# Telemedicine Preexposure Prophylaxis Prescribing From a Large Online US Company

**DOI:** 10.1001/jamanetworkopen.2025.46792

**Published:** 2025-12-01

**Authors:** Aaron J. Siegler, Shi Hao Ernest Koh, Tristan Schukraft, Simon Mazzoni, Edwin Corbin-Gutierrez, Rupa R. Patel, Patrick S. Sullivan, Wenting Huang

**Affiliations:** 1 Department of Epidemiology, Rollins School of Public Health, Emory University, Atlanta, Georgia; 2 MISTR LLC, San Juan, Puerto Rico; 3 National Alliance of State & Territorial AIDS Directors, Washington, DC; 4 Whitman-Walker Health, Washington, DC; 5 Division of Infectious Diseases, Washington University in St Louis, St Louis, Missouri

## Abstract

**Importance:**

HIV preexposure prophylaxis (PrEP) is highly effective in HIV prevention; telemedicine is a promising avenue for delivering PrEP care.

**Objective:**

To explore telemedicine delivery of PrEP care (telePrEP) by the largest telePrEP company in the US.

**Design, Setting, and Participants:**

This retrospective, single-arm cohort study used health record data of patients receiving telePrEP from MISTR LLC, a for-profit telehealth company, from November 27, 2018, to March 5, 2025. Patient characteristics and AIDSVu data were assessed to determine levels of PrEP use nationally. Retention in care was assessed for persons initiating PrEP in 2020, 2021, and 2022. Data were analyzed from March 5 to May 29, 2025.

**Exposure:**

Use of telePrEP.

**Main Outcomes and Measures:**

The primary outcome was the proportion of patients receiving telePrEP among all patients receiving PrEP in the US. Persons were classified as PrEP users if they received at least 1 day of prescribed PrEP in a calendar year.

**Results:**

From 2018 through 2025, 162 422 unique patients received telePrEP prescriptions from the telehealth company. Patients were predominantly young (mean [SD] age, 33.3 [10.3] years) and male (156 255 of 162 417 [96%]). Of 162 406 patients with data available, 19 610 (12%) were African American or Black, 12 075 (7%) were Asian, 39 744 (25%) were Hispanic or Latino, 82 222 (51%) were White, and 8755 (5%) were of other race or ethnicity. Most had not previously used PrEP (57 597 of 74 860 [77%]), and more than one-third (58 761 of 162 403 [36%]) were uninsured. Most users (131 875 [81%]) opted for home laboratory specimen collection as part of their care. TelePrEP use increased substantially from 6443 patients in 2020 to 110 068 in 2024. TelePrEP as a proportion of all national PrEP use was 2% in 2020, 9% in 2022, and 19% in 2024. Retention in care was similar for cohorts starting in 2020, 2021, and 2022; 67% (16 716 of 24 976) to 73% (4571 of 6287) were retained in the calendar year after initiation, 42% (8408 of 19 916) to 43% (2732 of 6287) in the second year, and 32% (2043 of 6287) to 33% (6546 of 19 916) in the third year. Annual care reinitiation ranged from 2% to 5%.

**Conclusions and Relevance:**

This cohort study found that nearly 1 in 5 PrEP users in the US received their PrEP prescriptions from a single national telemedicine company in 2024. Most were new to PrEP and more than one-third were uninsured. TelePrEP provision benefits from facilitating policies such as Affordable Care Act coverage and the 340B Drug Pricing Program. The rapid growth and overall magnitude of telePrEP provision suggests that it is a critical part of HIV prevention infrastructure in the US that merits continued regulatory support.

## Key Points

**Question:**

What are the provision characteristics and magnitude of preexposure prophylaxis care provided via telemedicine (telePrEP) in the US?

**Findings:**

This cohort study included 162 422 unique individuals who received telePrEP prescriptions from an online company from 2018 through 2025, most of whom had not previously used PrEP. TelePrEP as a proportion of total national PrEP use increased from 2% in 2020 to 19% in 2024.

**Meaning:**

These findings suggest that telePrEP has become a critical part of the HIV prevention infrastructure in the US, highlighting the importance of regulations supporting its provision.

## Teaser

This cohort study characterizes the use of telemedicine-delivered preexposure prophylaxis for HIV prevention in terms of its overall magnitude, user characteristics, changes in use over time, and retention in care.

## Introduction

HIV preexposure prophylaxis (PrEP) is a highly efficacious intervention that can prevent more than 99% of sexual HIV transmission when taken as prescribed.^1^ PrEP use has increased substantially in the US since its introduction; in 2023 more than 500 000 people used PrEP.^2^ However, this success still falls short of prevention targets; the Centers for Disease Control and Prevention (CDC) estimates that 2.2 million individuals would benefit from PrEP use.^3,4^ Moreover, disparities in PrEP use relative to HIV diagnoses (PrEP-need ratios) have emerged, with persistent lower PrEP-need ratios relative to overall levels among younger people, women, Black persons, and people in the US South.^5,6^ Numerous barriers exist to accessing PrEP and to staying engaged in PrEP care. One important barrier to retention in PrEP care is the clinical recommendation for at least 4 care visits annually for ongoing monitoring of HIV and sexually transmitted infections (STIs).^7^ The burden of seeking in-person care is considerable. Many PrEP users in the US do not live in close physical proximity to their PrEP prescribers; PrEP users in rural or suburban areas who seek care may face commute times that are greater than 1 hour round trip.^8^ Additional time demands are attributable to parking, waiting times, laboratory testing, the clinical visit itself, and prescription pickup at the pharmacy. Moreover, missed PrEP visits are associated with future discontinuation.^9^ By reducing transit and in-clinic wait time barriers, telemedicine is a promising avenue to facilitate PrEP access and retention in PrEP care.^10^

Telemedicine-delivered PrEP (telePrEP) has been evaluated as acceptable and feasible.^11,12,13,14^ Several ongoing studies plan to assess the efficacy of telePrEP in increasing PrEP initiation and retention in the US,^15^ the Netherlands,^15^ and Kenya.^16^ A randomized clinical trial assessing the impact of telePrEP found significantly higher self-reported PrEP initiations compared with standard of care at 90 and 180 days post enrollment, but no significant difference in self-reported PrEP adherence at either time point.^17^ The lack of higher PrEP adherence was also reported by a telePrEP demonstration study in Brazil.^18^

The provision of telePrEP is supported by public health infrastructure and regulations. State-level laws govern the provision and billing of telemedicine services. PrEP care requires frequent laboratory testing, including for HIV. The Affordable Care Act requires that prevention services, including telePrEP, are covered by most insurers. Last, the 340B Drug Pricing Program enables qualifying prescribers to receive purchase discounts on drugs from pharmaceutical companies, with returns able to be invested in care for uninsured patients, which allows access to PrEP care to be provided without regard to insurance coverage.

Several private companies offer telePrEP, but public evaluation data are limited: to our knowledge, no published studies have characterized the scale of this care modality. Using data from the largest national telePrEP company, MISTR LLC, we characterized the use of telePrEP in terms of its overall magnitude, user characteristics, changes in use over time, and retention in care.

## Methods

### Study Design and Data Source

We conducted a single-arm retrospective cohort study by analyzing electronic health record (EHR) data provided by the telehealth company from November 27, 2018 (when it began prescribing PrEP), until March 5, 2025. The data included all patients who sought PrEP care from the telehealth company during this period. Eligible patients had at least 1 EHR indicating a filled prescription of either combined emtricitabine and tenofovir disoproxil fumarate (FTC-TDF) or emtricitabine and tenofovir alafenamide (FTC-TAF) for PrEP. Fills are defined as PrEP prescriptions with either a prescription shipping date for delivery (466 228 of 539 508 [86%]) or confirmed prescription fill (for in-person pickup), data that the telehealth company receives from their pharmacy partners. This study involved a secondary analysis of data provided by the telehealth company that had been collected as part of their service provision. There was no direct interaction with human participants and the data were from a limited dataset. A waiver of Health Insurance Portability and Accountability Act (HIPAA) authorization and informed consent was reviewed and approved by the Emory University Institutional Review Board. This study followed the Strengthening the Reporting of Observational Studies in Epidemiology (STROBE) reporting guideline.

To provide context for the study, we searched PubMed for English-language articles published from January 1, 2012, to March 1, 2025, that included the terms *telePrEP* or *telemedicine PrEP*. Prior analyses of telePrEP provision were predominantly pilot studies and review articles. Pilot studies and review articles consistently identified telePrEP as feasible and acceptable.^11,12,13,14^ One randomized clinical trial assessed the impact of telePrEP, identifying a significant increase in self-reported PrEP initiation but no association with self-reported PrEP adherence at 90- and 180-day postbaseline assessments.^17^ No publications addressed the scale of telePrEP provision or presented observational clinical data regarding telePrEP that spanned multiple jurisdictions.

### TelePrEP Care Provision

Patients seeking care from the telePrEP company register with the platform and then complete a brief intake survey assessing domains of sociodemographic characteristics (age, sex, educational attainment, and race and ethnicity, including African American or Black [a group that has received lower levels of PrEP provision than anticipated by their HIV burden], Asian, Hispanic or Latino, White, and other race or ethnicity [including American Indian or Alaska Native, Middle Eastern, Native Hawaiian or Other Pacific Islander, and other race or ethnicity]), sexual risk factors informing PrEP indication, prior testing including for STIs and HIV, drug allergies, current PrEP use, recent HIV exposure that would indicate postexposure prophylaxis, and interest in doxycycline postexposure prophylaxis. The full text of the intake survey is provided in the eAppendix in Supplement 1. After the intake survey was complete, patients attended a required video telemedicine visit with a clinician. The video visit with the clinician became required nationally by the company in 2020 to comply with the most rigorous state laws; before this time, phone call visits were also an option in some states. During video visits, physicians discuss with patients how to take PrEP, adverse effects, and required laboratory testing. The standard care medication prescribed is FTC-TAF for male patients and FTC-TDF for female patients; clinicians make decisions about the prescribed regimen in consultation with the patient.

The telehealth company provides telePrEP care at no out-of-pocket cost to all users of this service and has not turned away uninsured users. The company’s model involves working closely with 65 nonprofit community-based health organizations that are 340B-covered entities. These organizations contract with the company to operate telePrEP services for their patients by using resources from insurance coverage and the 340B Drug Pricing Program. telePrEP services include video clinician visits, PrEP prescribing, contracted laboratory testing, patient support (including prior authorizations and insurance appeals), marketing to increase uptake of PrEP services, and doxycycline postexposure prophylaxis. For uninsured patients, the cost of their PrEP medication is covered by manufacturer’s patient assistance programs. For insured patients, the cost of copayments is usually covered by manufacturer copayment assistance programs. Medication costs of insured patients are covered by insurers at full cost, whereas the medication purchase price is discounted according to the 340B Drug Pricing Program. The difference between full coverage and discounted cost (eg, revenue) is used by the nonprofit community health organizations to cover the company’s telePrEP services. For all patients, including uninsured patients, the 340B funds received by covered entities are used in part to contract with the company. Neither the company nor the community health organization bill insurers for services provided regarding laboratory testing, telemedicine visits, or doxycycline postexposure prophylaxis provision; instead, the costs of this service provision are paid with 340B revenue.

Patients select initial HIV and STI tests at either a local in-person laboratory or remote laboratory testing. Remote testing involves a kit mailed to the patient’s home, a specimen self-collection procedure, and return mailing of specimens to a central laboratory for testing. Patients can select to pick up pills from a local pharmacy or have the prescription mailed to their home from a partner pharmacy. After laboratory results are received by the telePrEP company, the clinician makes a final PrEP eligibility determination. Patients whose HIV test results are reactive are linked to HIV care with a local clinician. Patients with reactive hepatitis B surface antigen test results are not eligible for telePrEP from this company and are encouraged to seek PrEP from a local clinician; local clinicians can offer closer monitoring of hepatitis B virus infection in the setting of PrEP medication.

### Statistical Analysis

Descriptive data (eg, sociodemographic characteristics and sexual behaviors) were collected by the telePrEP company in a HIPAA-compliant electronic, self-reported survey at intake. Data on prescriptions and laboratory test orders were obtained from EHRs.

Data were analyzed from March 5 to May 29, 2025. To calculate the proportion of telePrEP prescriptions among all national PrEP prescriptions, we used annual national PrEP prescription data provided on the AIDSVu site that uses a dataset that is agnostic to modality, meaning that it includes both telePrEP and standard PrEP prescribing.^6,19^ We calculated periods of PrEP use that align with the AIDSVu method that has been used by the Centers for Disease Control and Prevention: a person with at least 1 day of PrEP prescribed during a calendar year was determined to be a PrEP user during that period.^5,20^ This metric includes days of prescribed PrEP accumulated from the prior period that would be expected to be used in the next period if used with daily dosing. No inferential statistics were performed, as this study is a near-census data analysis and the data included all users from the largest telePrEP company in the US, comprising 20% of all US PrEP users.

## Results

### Characteristics of Patients Receiving TelePrEP

**Figure 1.**
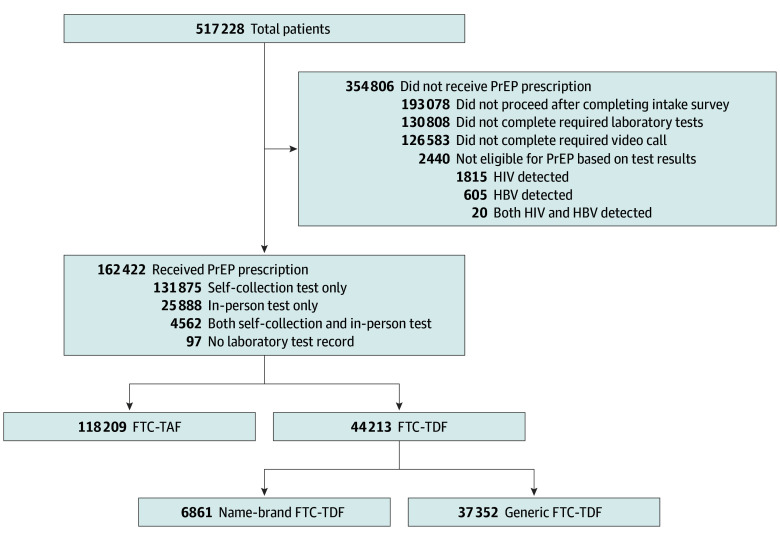
Flowchart of Patients From Intake to Preexposure Prophylaxis (PrEP) Prescription The telemedicine company requires completion of an intake survey, laboratory testing results, and a video telemedicine visit. For laboratory testing, patients could go to an in-person venue or receive a mailed kit with self-collection materials. PrEP prescriptions included combined emtricitabine and tenofovir disoproxil fumarate (FTC-TDF) or emtricitabine and tenofovir alafenamide (FTC-TAF). All FTC-TAF prescriptions were name brands. HBV indicates hepatitis B virus.

Overall, the telePrEP company recorded interactions with 517 228 patients on their electronic platform from November 2018 to March 2025, of whom 162 422 (31%) received at least 1 PrEP prescription (Figure 1). Several components of care are required by the telePrEP company for initiation. Among those patients not initiating PrEP, 193 078 (54%) did not interact with the company after their required intake survey. Among persons who proceeded after the intake survey, more than three-quarters did not have the required online video visit with a telemedicine clinician (126 583 [78%]) or did not complete the required laboratory tests (130 808 [81%]). Twenty persons (1%) were ineligible for PrEP based on HIV or hepatitis B surface antigen test results. Among the 162 422 patients who obtained a PrEP prescription, 131 875 (81%) received the remote laboratory tests. A total of 118 209 patients (73%) received their initial prescriptions for FTC/-TAF, and 44 213 (27%) received prescriptions for FTC-TDF. Most of the patients receiving FTC-TDF (37 352 [85%]) received a generic formulation.

The Table shows the descriptive characteristics of patients using telePrEP who received a PrEP prescription. The mean (SD) age was 33.3 (10.3) years, with 156 255 patients (96%) being male at birth, 6162 (4%) female, and data missing for 5 (<1%). A total of 121 011 patients (75%) identified as gay and 29 072 (18%) identified as bisexual. For race and ethnicity, 19 610 patients (12%) were African American or Black, 12 075 (7%) were Asian, 39 744 (25%) were Hispanic or Latino, 82 222 (51%) were White, and 8755 (5%) were of other race or ethnicity. Of 157 618 patients with data available, 75 735 (48%) had a bachelor’s or a master’s degree, and 58 761 of 162 403 (36%) were uninsured. In terms of behavioral characteristics in the last 6 months, 125 941 of 162 381 patients with data available (78%) reported condomless anal sex and 14 072 of 162 380 (9%) reported at least 1 STI diagnosis in the previous 6 months. Of 74 860 patients with data available, 57 597 (77%) had never taken PrEP before completing the intake survey.

**Table.** Sociodemographic and Behavioral Characteristics of Patients Receiving Telemedicine Delivery of PrEP, 2018 to 2025^a^

Characteristic	No. (%) of patients (N = 162 422)
Age, mean (SD), y	33.3 (10.3)
Age range, y	
<19	1307 (1)
19-30	74 723 (46)
31-40	54 726 (38)
41-50	18 866 (12)
>50	12 800 (8)
Sex	
Male	156 255 (96)
Female	6162 (4)
Sexual orientation	
Gay	121 011 (75)
Bisexual	29 072 (18)
Straight	6589 (4)
Prefer not to answer	5735 (4)
Race and ethnicity	
African American or Black	19 610 (12)
Asian	12 075 (7)
Hispanic or Latino	39 744 (25)
White	82 222 (51)
Other^b^	8755 (5)
Educational attainment	
High school or less	29 680 (19)
Some college, associate’s degree, or technical school	52 203 (33)
Bachelor’s degree	52 858 (34)
Master’s or professional degree	22 877 (15)
Annual household income	
<$20 000	12 694 (22)
$20 000-$29 999	13 666 (23)
$30 000-$39 999	11 842 (20)
≥$40 000	20 853 (35)
Health insurance coverage	
Yes	103 642 (64)
No	58 761 (36)
Condomless sex^c^	
Yes	125 941 (78)
No	33 734 (21)
Not sure	2706 (2)
Any HIV-positive sexual partners^c^	
Yes	8878 (6)
No	121 985 (75)
Not sure	31 517 (19)
Prior STI diagnosis^c^^,^^d^	
Yes	14 072 (9)
No	148 308 (91)
Currently taking PrEP	
Yes	34 277 (21)
No	128 108 (79)
Ever taken PrEP before survey	
Yes	17 263 (23)
No	57 597 (77)

Abbreviations: PrEP, preexposure prophylaxis; STI, sexually transmitted infection.

^a^ Different levels of completion were due to survey version updates over time and some questions being optional. Numbers for certain characteristics may not sum to the total number of patients owing to missing data.

^b^ Includes American Indian or Alaska Native, Middle Eastern, Native Hawaiian or Other Pacific Islander, and other race or ethnicity.

^c^ Indicates within the last 6 months.

^d^ Self-reported STIs included syphilis, chlamydia, and gonorrhea.

### TelePrEP Use Over Time

**Figure 2.**
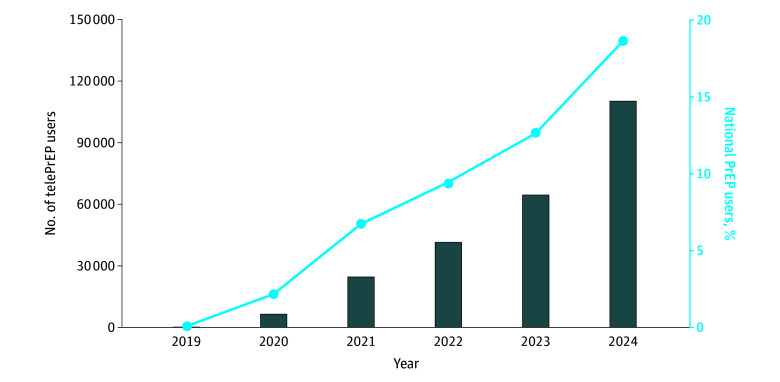
Absolute Number and National Proportion of Telemedicine Preexposure Prophylaxis (TelePrEP) Users Data from 2018 and 2025 are not shown because telePrEP accounted for less than 0.01% of prescribing in 2018 and 2025 data are incomplete (encompassing January through March only).

There were 204 telePrEP users in 2019, 41 453 users in 2022, and 110 068 users in 2024 (Figure 2). As a proportion of all estimated US PrEP users, in 2019 telePrEP use was less than 1 in 100 PrEP users; in 2022, approximately 1 in 10; and in 2024, approximately 1 in 5.

### TelePrEP Retention in Care

**Figure 3.**
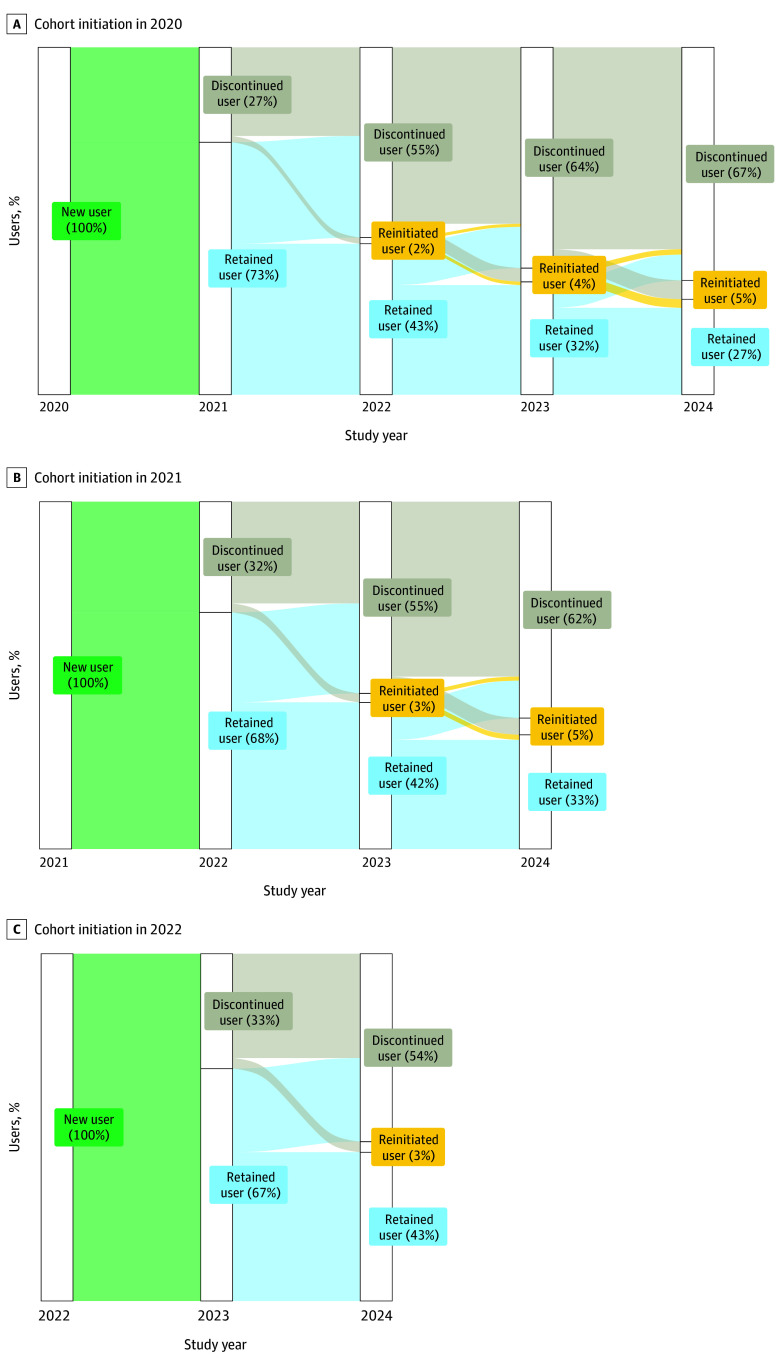
Annual Telemedicine Preexposure Prophylaxis (PrEP) Retention and Reinitiation by Calendar Year of Initiation Cohort initiation is shown for 2020 (6287 patients), 2021 (19 916 patients), and 2022 (24 976 patients).

Figure 3 shows annual retention in PrEP care for persons initiating PrEP in calendar years 2020, 2021, and 2022. There were similar levels of retention among cohorts stratified by calendar year of PrEP initiation. In the first calendar year after PrEP initiation, 67% (16 716 of 24 976) to 73% (4571 of 6287) of users were retained in care. In the second year after initiation, 42% (8408 of 19 916) to 43% (2732 of 6287) of users were retained in care and 2% (113 of 6287) to 3% (759 of 24 976) of users reinitiated care. In the third year after initiation, 32% (2043 of 6287) to 33% (6546 of 19 916) of users were retained in care and 4% (249 of 6287) to 5% (964 of 19 916) reinitiated care. In the fourth year after initiation (2020 PrEP starts only), 27% (1726 of 6287) were retained in care and 5% (341 of 6287) reinitiated care.

## Discussion

In the first national assessment of telePrEP provision, to our knowledge, we estimated that 1 in 5 PrEP users in 2024 received their PrEP prescriptions from a single national telePrEP company. This magnitude—more than 110 000 users in 2024—indicates the role for telemedicine in achieving scale and impact for a highly effective HIV prevention service. This is an underestimate of total telePrEP use because our data represent a single company and there are other telePrEP companies. Since 2019, our data indicate a rapid growth in telePrEP use both in terms of the absolute number of users and the proportion of telePrEP use as a share of total national PrEP use. This growth was not predominantly among users switching from traditional PrEP care to telemedicine; instead, more than three-quarters of users had never previously used PrEP.

Nearly one-third of telePrEP users were uninsured, a higher share than would be expected given that fewer than one-tenth of people in the US lack health insurance.^21^ High levels of service provision to people without insurance is facilitated by manufacturer payment assistance programs and 340B Drug Pricing Program that provides funds to collaborating community-based health organizations for such service coverage; this also indicates the critical nature of the 340B program for highly accessible PrEP provision. The high accessibility of telePrEP to people without insurance is notable, because previous studies have identified uninsured status to be associated with HIV incidence.^22,23^ Patients receiving telePrEP were relatively similar to the overall US population in terms of race and ethnicity.^24^ Compared with their national population share of new HIV diagnoses (Black and Hispanic or Latino persons accounted for 39% and 31% of diagnoses, respectively); however, there were lower levels of PrEP use by Black and Hispanic or Latino persons (14% and 18% of PrEP prescriptions, respectively).^6^ Similar differences in PrEP use are seen across PrEP prescribing modalities nationally, as previously described with PrEP-need ratios.^5,20,25^

Most users opted for home HIV and STI testing instead of in-person testing options, and such strong preference highlights the importance of this avenue for a user group that is predominantly young (nearly half aged ≤30 years). Younger people have much lower use of primary care,^26^ and remote services offer an attractive mechanism to bridge this gap. Remote screening has been an area of increased regulatory attention, with remote blood specimen collection for HIV screening that previously had been allowed to operate under a laboratory-determined test protocol now being considered a device requiring approval based on 2024 communication from the US Food and Drug Administration (FDA).^27^ This communication classified dried blood spot and microtainers that have been used to collect specimens as a device that would require formal FDA approval. Previously, the use of dried blood spots and microtainer blood collection was covered under the Clinical Laboratory Improvement Amendments (CLIA) laboratory validation process. Additional validation assessments as suggested by FDA regulation may have long-term benefits to ensure high-quality testing protocols and services. To the extent that having at-home testing options facilitate ongoing engagement in PrEP care, the public health benefit of the current approval process with CLIA validation should be given consideration in the evaluation of the net benefits of self-collection laboratory services. Given that 131 875 users have used home testing with PrEP, it is important to find ways to ensure that avenues for remote testing remain open for health care access until a more thorough assessment of validity can be made with device approval.

In absolute terms, retention in care was suboptimal over time, with fewer than one-third of users discontinuing after 1 calendar year and more than half discontinuing after 2 years. In relative terms, however, retention was higher than observed in general PrEP care from several cohorts in the US.^28,29^ Outside telemedicine, studies have identified a range of factors contributing to limited retention, including stigma,^30^ lower perceived risk,^31^ clinic logistics or scheduling barriers,^32^ and medication concerns such as current or long-term adverse effects.^32^ Further assessment is needed regarding retention in care for telePrEP. Retention in telePrEP care had remarkably little variation over time, with a difference of only a few percentage points across 3 different cohorts grouped by year of initiation (2020, 2021, and 2022). This stability indicates an area for potential improvement; future efforts could seek to identify and strengthen company- or system-level factors associated with telePrEP care retention and optimize these factors to support high retention.

Nearly three-quarters of PrEP prescriptions were for FTC-TAF, with the remaining one-quarter of prescriptions being FTC-TDF. Patient-preferred PrEP modalities are ones that have lower adverse effect profiles, less frequent dosing intervals, and coverage from insurers.^33,34^ Some clinicians prefer FTC-TAF,^35^ possibly for reasons relating to adverse effects, although the relative difference in adverse effect burden between FTC-TAF and FTC-TDF has been debated. There have been calls to increase the prescribing of less expensive, generic versions of PrEP to save overall health care costs.^36^ As newer long-acting PrEP modalities are under development and testing, we anticipate there will be a data-informed debate regarding which modalities of PrEP to scale that must consider the balance of patient and clinician preferences, relative health benefits of different modalities, and medication costs.

There were low levels of PrEP care reinitiation over time among people who stopped their initial PrEP prescription: only 2% to 5% of persons reinitiated PrEP care after discontinuing. A study in Connecticut^37^ found that PrEP reinitiation was common when using a PrEP care reinitiation definition of greater than 1 month without refill as a discontinuation event. When defining reinitiation as restarting after longer than 1 month without a refill, lower levels of reinitiation were observed. We recommend further assessments that incorporate multiple periods of discontinuation; such analyses would yield deeper insights into reinitiation patterns.

### Limitations

This study has several limitations. Although several US companies offer telePrEP care and other companies offer telePrEP care locally, we used data from only a single telePrEP company. We were unable to obtain data from other national telehealth companies, and such agreements were not feasible to negotiate for the large potential number of local companies. This selection bias almost certainly resulted in an underestimation of national telePrEP use. National PrEP use data estimations (the denominator for the calculation of proportion of all US PrEP provided through telePrEP) rely on national prescription data and may not include all claims,^20^ potentially leading to overestimation of the proportion of telePrEP use relative to national PrEP use. The electronic medical record system of the telePrEP company changed over time, resulting in missing data.

## Conclusions

This cohort study found that in 2024, almost 20% of PrEP users in the US received their PrEP prescriptions from a single national telemedicine company. TelePrEP has become a major source for PrEP care in the US. It has substantial adoption by populations with limited health care access and by groups who previously had limited PrEP utilization. Retention in PrEP care remains a challenge—as it does in clinic-based PrEP care—and retention should be targeted for improvement. Ensuring higher levels of uptake among groups most at risk for HIV remains a challenge for both in-person PrEP and telePrEP modalities. The large scale of telePrEP, with more than 100 000 users in 2024, indicates the central importance of maintaining and expanding this route of accessing a highly effective prevention modality.

## Notes

Supplement 1. **eAppendix.** MISTR Intake Questionnaire

Supplement 2.
Data Sharing Statement
