# Supplementary material for: Telemedicine Preexposure Prophylaxis Prescribing From a Large Online US Company
Source: JAMA Netw Open. 2025 Dec 1;8(12):e2546792. doi: 10.1001/jamanetworkopen.2025.46792 (PMC12670195; doi:10.1001/jamanetworkopen.2025.46792)
Supplement: Supplement 1. — eAppendix. MISTR Intake Questionnaire [file jamanetwopen-e2546792-s001.pdf]

Supplementary Online Content

Siegler AJ, Koh SHE, Schukraft T, et al. Telemedicine preexposure prophylaxis prescribing from a large online US company. *JAMA Netw Open.* 2025;8(12):e2546792. doi:10.1001/jamanetworkopen.2025.46792

eAppendix. MISTR Intake Questionnaire

This supplementary material has been provided by the authors to give readers additional information about their work.

© 2025 Siegler AJ et al. *JAMA Network Open*.

eAppendix. MISTR Intake Questionnaire

1. Sex assigned at birth?*
    - Male
    - Female

2. Gender identity?*
    - Male
    - Female
    - Transgender Male
    - Transgender Female
    - Gender Queer
    - Gender Nonconforming

3. Preferred pronouns?*
    - He/him/his
    - She/her/hers
    - They/them/their

4. Date Of Birth* ______________

5. Height?* ______________

6. Weight (lb)? ______________

7. How do you consider yourself?*
    - Gay
    - Bisexual
    - Straight
    - I prefer not to answer

8. What race / ethnicity do you most closely identify with?*
    - African American / Black
    - American Indian or Alaska Native
    - Asian
    - Caucasian / White
    - Hispanic or Latino
    - Middle Eastern
    - Native Hawaiian or Pacific Islander
    - Other

9. What is the highest degree or level of school you have completed?*
    - Some high school, no diploma
    - Trade / technical / vocational training
    - Some college, no degree
    - High school diploma or equivalent
    - Associate degree (e.g. AA, AS)
    - Bachelor's degree (e.g. BA, BS)
    - Master's degree (e.g. MA, MS, MEd)
    - Professional degree (e.g. MD, DDS, DVM)

© 2025 Siegler AJ et al. *JAMA Network Open*.

10. Annual household income? ______________

11. Number of people in household? ______________

12. What is your current relationship status?*
    - Single
    - Partnered, committed relationship
    - Partnered, open relationship
    - Married, or in a domestic partnership
    - Married / Partnered, committeed relationship
    - Married / Partnered, open relationship

13. Are you HIV positive? (ALERT IF YES)
    - Yes
    - No

14. Have you ever been diagnosed with AIDS (not just HIV infection)?
    - Yes
    - No

15. What medication are you currently taking to treat your HIV?
    - Atripla (efavirenz + tenofovir disoproxil fumarate + emtricitabine)
    - Biktarvy (bictegravir + tenofovir alafenamide + emtricitabine)
    - Cabenuva (cabotegravir + rilpivirine)
    - Complera (rilpivirine + tenofovir disoproxil fumarate + emtricitabine)
    - Delstrigo (doravirine + tenofovir disoproxil fumarate + lamivudine)
    - Dovato (dolutegravir + lamivudine)
    - Genvoya (elvitegravir + cobicistat + tenofovir alafenamide + emtricitabine)
    - Juluca (dolutegravir + rilpivirine)
    - Odefsey (rilpivirine + emtricitabine + tenofovir alafenamide)
    - Stribild (elvitegravir + cobicistat + tenofovir disoproxil fumarate + emtricitabine)
    - Symfi and Symfi Lo (efavirenz + tenofovir disoproxil fumarate + lamivudine)
    - Symtuza (darunavir + cobicistat + tenofovir alafenamide + emtricitabine)
    - Triumeq (dolutegravir + abacavir + lamivudine)
    - Other (please specify)

16. Are you currently taking your medication everyday?
    - Yes
    - Sometimes I miss dose
    - No

17. When was the last time you saw a doctor and completed labs?*
    - 3 months
    - 6 months
    - 9 months
    - 12 months
    - More than 12 months

© 2025 Siegler AJ et al. *JAMA Network Open*.

18. Do you know what your most recent viral load was?
    - Yes
    - No

19. Do you know what your last CD4 count was?
    - Yes
    - No

20. Have you had your bone density checked within the last 5 years?
    - Yes
    - No

21. When was the last time you were tested for HIV?
    - Less than 30-days
    - 2-3 months
    - 3-6 months
    - 6-12 months
    - More than 12 months
    - Never been tested

22. Are you currently taking PrEP?
    - Yes
    - No

23. Have you taken PrEP before?*
    - Yes
    - No

24. Last time you were actively taking PrEP? *
    - less than 3 months
    - 3-6 months
    - 6-12 months
    - over 12 months
    - not sure

25. In the last 72-hours, have you been exposed to the semen or blood of someone you know to be HIV positive?* (ALERT IF YES)
    - Yes
    - No

26. How long has it been?* (ALERT IF PREVIOUS OPTION IS YES)
    - Less than 24-hours
    - More than 24-hours

27. Who are you normally having sexual intercourse with? Select all that apply.*
    - Men
    - Women
    - Transgender Partners

© 2025 Siegler AJ et al. *JAMA Network Open*.

28. In the last six months, how many sexual partners have you had?* ______________

29. What type of sex do you normally have?*
    - Anal (bottom)
    - Anal (top)
    - Oral
    - Vaginal
    - I Prefer not to answer

30. In the last 6 months, have you had any condomless sex?*
    - Yes
    - No
    - I'm not sure

31. In the last 6 months, have any of your sexual partners been HIV positive?*
    - Yes
    - No
    - I'm not sure

32. In the next 6 months, do you plan to have any of the following? Select all that apply.
    - Condomless sex
    - HIV positive partner
    - I'm not sure

33. Have you been diagnosed with any of the following in the last six months? Select all that apply.* **(ALERT IF CHLAMYDIA / GONORRHEA IS CHOSEN)**
    - Chlamydia
    - Gonorrhea
    - None of these

34. Have you ever been diagnosed with syphilis (in your lifetime)?* (ALERT IF YES)
    - Yes
    - No

35. Approximately how long ago were you diagnosed? (ALERT IF PREVIOUS OPTION IS YES)
    - Less than 3-months
    - 3-6 months
    - 6-12-months
    - 12+ months
    - I'm not sure

36. Have you received or are you receiving treatment?
    - Yes
    - No

37. Have you ever been diagnosed with any of these conditions? Select all that apply.* **(ALERT IF ANY OPTION EXCEPT 'NONE OF THESE' IS CHECKED)**
    - Hepatitis B
    - Hepatitis C
    - Kidney Disease

© 2025 Siegler AJ et al. *JAMA Network Open*.

- Uncontrolled Diabetes
- Liver Disease
- None of these

38. Are you currently taking any prescription medications?
    - Yes
    - No

39. Are you currently taking any herbal treatments like St. John's Wort or nutritional supplements such as creatine?
    - Yes
    - No

40. Do you have drug allergies?* (ALERT IF YES)
    - Yes
    - No

41. Please list your drug allergies and explain what happens when you use that drug.* (ALERT IF PREVIOUS IS YES) ______________

42. Do you have any questions or is there anything else we should know?* (ALERT IF YES)
    - Yes
    - No

43. Are you aware of Doxy PEP?
    - Yes
    - No

44. Are you allergic to tetracycline or doxycycline or are you currently on oral retinoids such as Accutane, or Retin-A/tretinoin?
    - Yes
    - No

45. Are you interested in receiving Doxy PEP at no cost?
    - Yes
    - No

46. Do you have insurance?
    - Yes
    - No

47. Testing type preference?
    - In person
    - At home

© 2025 Siegler AJ et al. *JAMA Network Open*.
