# Supplementary material for: Telemedicine Preexposure Prophylaxis Prescribing From a Large Online US Company
Source: JAMA Netw Open. 2025 Dec 1;8(12):e2546792. doi: 10.1001/jamanetworkopen.2025.46792 (PMC12670195; doi:10.1001/jamanetworkopen.2025.46792)
Supplement: Supplement 2. — Data Sharing Statement [file jamanetwopen-e2546792-s002.pdf]

Data Sharing Statement

Siegler. Telemedicine Preexposure Prophylaxis Prescribing From a Large Online US Company. *JAMA Netw Open*. Published December 08, 2025.
doi:10.1001/jamanetworkopen.2025.46792

Data
Data available: No

Additional Information
Explanation for why data not available: Proprietary health data were provided for this analysis under a data sharing agreement between MISTR LLC and Emory University. Data for this publication are not available for public sharing at this time.
